# Xenograft models of head and neck cancers

**DOI:** 10.1186/1758-3284-1-32

**Published:** 2009-08-13

**Authors:** Daisuke Sano, Jeffrey N Myers

**Affiliations:** 1 Department of Head and Neck Surgery, The University of Texas M. D. Anderson Cancer Center, Houston, Texas, USA

## Abstract

Head and neck cancers are among the most prevalent tumors in the world. Despite advances in the treatment of head and neck tumors, the survival of patients with these cancers has not markedly improved over the past several decades because of our inability to control and our poor understanding of the regional and distant spread of this disease. One of the factors contributing to our poor understanding may be the lack of reliable animal models of head and neck cancer metastasis. The earliest xenograft models in which human tumor cells were grown in immunosuppressed mice involved subcutaneous implantation of human head and neck cancer cell lines. Subcutaneous xenograft models have been popular because they are easy to establish, easy to manage, and lend themselves to ready quantitation of the tumor burden. More recently, orthotopic xenograft models, in which the tumor cells are implanted in the tumor site of origin, have been used with greater frequency in animal studies of head and neck cancers. Orthotopic xenograft models are advantageous for their ability to mimic local tumor growth and recapitulate the pathways of metastasis seen in human head and neck cancers. In addition, recent innovations in cell labeling techniques and small-animal imaging have enabled investigators to monitor the metastatic process and quantitate the growth and spread of orthopically implanted tumors. This review summarizes the progress in the development of murine xenograft models of head and neck cancers. We then discuss the advantages and disadvantages of each type of xenograft model. We also discuss the potential for these models to help elucidate the mechanisms of regional and distant metastasis, which could improve our ability to treat head and neck cancers.

## Introduction

Head and neck cancers consistently rank among the six most frequently diagnosed cancers in the world. Cancers of the oral cavity and pharynx alone account for 337,931 new cases worldwide and 183,613 deaths annually [1]. In 2008, 35,310 new head and neck cancers were diagnosed in the United States, representing approximately 2.5% of all cancers diagnosed [2]. Over 90% of head and neck cancers are squamous cell carcinomas of the upper aerodigestive tract, including the oral cavity, pharynx, larynx, and paranasal sinuses. In addition, epithelial head and neck tumors can arise in the salivary and thyroid glands. Despite advances in our understanding and advances in the prevention and treatment of head and neck cancers, the survival of patients with head and neck cancers has not significantly improved over the past several decades. Frequently, treatment failure takes the form of local and regional recurrences. Our limited understanding of the mechanisms of local and regional metastasis of these tumor types thus accounts for the poor prognosis for patients with head and neck cancers.

Although no animal model is perfectly applicable to every kind of human cancer, it is generally agreed that using preclinical animal xenograft tumor models is useful for modeling the growth and spread of disease and for acquiring information about the mechanisms of action and therapeutic efficacy of new antitumor agents. The literature is replete with articles documenting cancer biology and preclinical evaluations of anticancer agents in xenograft models for human cancers.

Animal models are being used with greater frequency to advance our understanding of the mechanisms of regional and distant metastatic spread of head and neck cancers. This review summarizes the progress in the development of murine xenograft models of head and neck cancers. These models can help elucidate the mechanisms of regional and distant metastasis to improve our ability to treat head and neck cancers.

### Subcutaneous xenograft models

In 1955, the National Cancer Institute (NCI) began to use mouse a models bearing rapidly growing murine leukemia cells that had been injected intraperitoneally for systematic drug screening. These models were successful in identifying the effect of therapeutic agents against hematologic malignancies, but they were not as useful for devoloping agents to treat solid tumors. Xenograft studies of solid tumors received a major boost in 1971 with the observation that the athymic nude mice could be used to establish and grow human tumors [3]. Athymic mice lack mature T-cells, which are believed to be involved in tumor immune surveillance and are critical to "self" recognition and destruction of grafted non-self tissues. This loss of T-cell function enables cross-species "xenografted" tissues, including tumor cells, to be tolerated by the immune system of the recipient animal.

Most preclinical studies that have been performed using xenografts tumor models have used subcutaneous implantation of tumor cells. Several studies have reported that subcutaneous xenograft models can predict the clinical activity of cytotoxic agents [4-6]. However, other studies have provided an opposing view. The National Cancer Institute, NCI retrospectively reviewed 39 agents that had been studied preclinically using subcutaneous xenograft models and in phase II clinical trials in human patients. This analysis revealed that the *in vivo *anti-tumor activity in the preclinical animal models did not closely correlate with therapeutic response in human cancers of the same histology, except in non-small-cell lung cancers. [7].

Subcutaneous tumor models are advantageous because of their ease of tumor establishment, measurement, and reproducibility [8]. However, ectopic subcutaneous xenograft models are less useful for studying agents that modulate the tumor microenvironment, as an ectopic site does not reproduce the primary tumor site microenvironment as well as an orthotopic site does. This rationale has been used to explain why many therapeutic compounds that have shown promising activity in subcutaneous xenograft models revealed disappointing results when tested in clinical trials [9]. The difference between the drug activity in preclinical trials and the activity in clinical trials might be related to the treatment of advanced and/or metastatic disease in the clinic. Conventional subcutaneous xenograft models do not recapitulate advanced local-regional or distant metastatic disease [10], as they do not have organ-specific environments for metastatic tumor cells nor do they represent the common sites of metastasis. For examples, Sharkey and Fogh [11] reported only a 1.3% incidence of metastasis in the subcutaneous site in a total of 1,045 nude mice involving 11 different tumor lines.

Xenografts models of head and neck cancers have been published since the early 1980s. In 1984, Braakhuis et al. [12] reported on their xenografts model of head and neck cancer, which were established from fresh surgical specimens from 130 human head and neck cancers implanted into the subcutaneous tissues of nude mice. Tumor growth was observed in 26% of the mice, and the highest rate of growth was observed in animals with poorly differentiated, metastatic, and hypopharyngeal tumors. In another study, Baker reported that 16 of the 21 squamous cell carcinoma cell lines (76%) that were heterotransplated into nude mice developed viable tumor nodules. In these articles, no evidence of metastasis was demonstrated [13].

### Orthotopic xenograft model

One of the first reports on orthotopic xenograft models, published by Tan et al. in 1977, established orthotopic transplantation of murine colon adenocarcinomas into the colon of syngeneic mice, which led to an increase in hepatic metastases compared to that seen with ectopic injection [14]. This study by Tan et al. and other studies have found that orthotopic xenograft models can represent a more clinically relevant tumor model with respect to the tumor's primary site and metastasis [8]. In orthotopic xenograft models, host microenvironments are more closely mimicked by implanting tumor cells into the original anatomical sites when compared to subcutaneous xenograft models. Some studies have noted differences in biological behavior when tumors are grown subcutaneously relative to tumors grown orthotopically. Organ-specific environments are also useful for evaluating anti-tumor therapeutics, as the sensitivity of xenografted tumors to drugs and/or radiation may be modulated by their location, and metastasis may be influenced by the tumor implant sites. One of the advantages of the orthotopic model is that orthotopic implantation of tumor cells can result in high rates spontaneous tumor metastasis, whereas the same cells implanted subcutaneously rarely metastasize [10]. Another advantage of the orthotopic system is that attempts to target the processes involved in local invasion (e.g., inhibition of proteases or interfering with angiogenesis) can be carried out in a more clinically relevant site [15]. Finally, some investigators have described the differences between preclinical drug activity in subcutaneous and orthotopic models and reported that orthotopic models are more appropriate for predicting clinical response [15,16].

Orthotopic xenograft models have some disadvantages as well. For example, these models can be more technically challenging to establish and may cause animal morbidity and even death. Also, for internal, poorly accessible orthotopic sites, it can be difficult to evaluate antitumor efficacy in a continuous way. However, the use of noninvasive methods to measure tumor volume, including small-animal magnetic resonance imaging; positron emission tomography; reporter genes with specific fluorescence properties, such as the stable green fluorescent protein [17,18]; and the luciferase gene [19], helped to overcome these obstacles to accurately measure internally implanted orthotopic tumors [15,20].

### Orthotopic xenograft model of head and neck cancers

Orthotopic xenograft models of head and neck squamous cell carcinomas (HNSCCs) have been reported since the late 1980s [21-24]. In one of these studies, Fitch et al. [21] aspirated cells from subcutaneous xenograft models made from fresh human tumors in nude mice and then injected these cells into the tongues of nude mice. The authors reported an 86% of incidence of these implanted oral tumors [21]. Dinesman et al. [22] implanted tumor cells into the floor of the mouth through a submandibular route in nude mice. They reported that 5% of their mice had lymph node metastases and 40% had pulmonary metastases, which supports the hypothesis that orthotopic xenograft models effectively reproduce the patterns of tumor metastasis seen in human patients. Simon et al. have reported another floor-of-mouth invasion xenograft model [24].

Kawashiri et al. reported on an orthotopic sublingual model of squamous cell carcinoma of the oral tongue (SCCOT) that had a high incidence of cervical lymph node metastases [23,25]. Myers et al. also established orthotopic models of SCCOT through injection of human cell lines into the oral tongue of nude mice, which led to the development of cervical lymph node and pulmonary metastasis [26]. In that article, the authors found that serial passage of the lymph nodes by isolating the regional metastases from the cervical lymph nodes after the development of orthotopic tongue tumors resulted in a cell line that was more metastatic than its parental line. Myers et al. also showed that the tumorigenicity of oral cancer cells was greater when the cells were injected into the tongue rather than under the skin of nude mice. Qui et al. implanted fresh lymph node metastatic human specimens into the tongues of nude mice to establish their orthotopic model of tongue [27]. These authors reported that serial passage of the lymph nodes led to the development of cell lines that have more potential to metastasize to the cervical lymph nodes.

**Figure 1:**
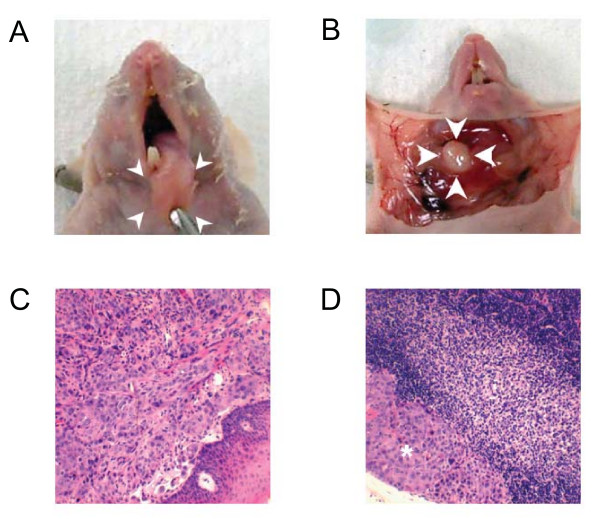
**Tongue tumor and cervical lymph node metastasis of SCCOT orthotopic xenografts**. (A) Primary tongue tumor after orthotopic sublingual implantation of OSC-19 cells isolated from a patient with a well-differentiated squamous cell carcinoma. The tongues of nude mice were inoculated with OSC-19 cells, and mice were sacrificed after 35 days. Necropsy was then performed and an obvious tumor was seen in the tongue. (B) Regional metastasis from orthotopic sublingual implantation of luciferase-transduced OSC-19 LN2-Luc cells. OSC-19 LN2-Luc cells were injected into the tips of the mice's tongues. Thirteen days after cell inoculation, the primary tumor was removed surgically. On day 82, increased intensity of photon emission from the cervical lymph node metastasis was observed. (C) and (D) Hematoxyilin and eosin slides of the OSC-19 tongue tumor and cervical lymph node of OSC-19 revealed squamous cell carcinoma in locations (*).

Because the oral tongues of the animals can be approached relatively easily, it is not very complicated to establish an orthotopic model of oral cancers. In brief, at our institution, mice are anesthetized, and the tongue is exposed by grasping it in the midline with a small-toothed forceps. Then 1 × 10^3 ^to 1 × 10^6^, head and neck squamous cancer cells are injected into the tongue submucosally using a 1-ml tuberculin syringe with a 30-gauge hypodermic needle. The orthotopic oral tongue cancers in the nude mouse resemble human SCCOT histologically (Figure 1). This techniques is relatively easy to establish and leads to cervical lymph node metastasis, and therefore, has many potential applications, such as studying the systemic cellular and molecular mechanisms of tumorigenicity, growth, and metastasis of HNSCC and assessing the effect of novel therapeutic regimens for SCCOT [28-30].

Only a few studies have been published on orthotopic models for other head and neck cancers. For example, Kim et al. [31] reported on an orthotopic model for thyroid carcinoma using anaplastic thyroid carcinoma cell lines. The authors showed that the tumorigenicity of thyroid cancer cells was greater when the cells were injected into the thyroid gland rather than under the skin of nude mice. Ahn et al. [32] also established an orthotopic model of papillary thyroid carcinoma in nude mouse using a technique similar to the technique used by Kim et al[31]. These thyroid orthotopic mouse models have been used to determined the effect of novel molecularly targeted agents for aggressive thyroid cancers [33-36].

In another study, Gelbard et al. reported the establishment of an orthotopic mouse model of sinonasal malignancy with a human epidermoid tumor cell lines injected into the right maxillary sinus or soft palate in nude mice [37]. Finally, Younes et al. established an orthotopic model of salivary cancer in the parotid glands of nude mice and reported treatment efficacy with an epidermal growth factor receptor/vascular endothelial growth factor receptor-targeted agent [38]. This same model was also reported by Choi et al. [39].

Although it is difficult to monitor tumor growth and metastasis spread in orthotopc models, noninvasive imaging methods have been useful for visualizing primary tumors and cervical lymph node metastasis in orthotopic models of head and neck cancers. For example, the green fluorescent protein has been used to image primary oral squamous cell carcinoma and metastasis [26,40] and bioluminescence with the enzyme, firefly luciferase, has been used for noninvasive *in vivo *tumor measurement. For this bioluminescence system, tumor cells need to be transfected with the luciferase gene, and are then given the substrate luciferein prior to imaging [41].

**Figure 2:**
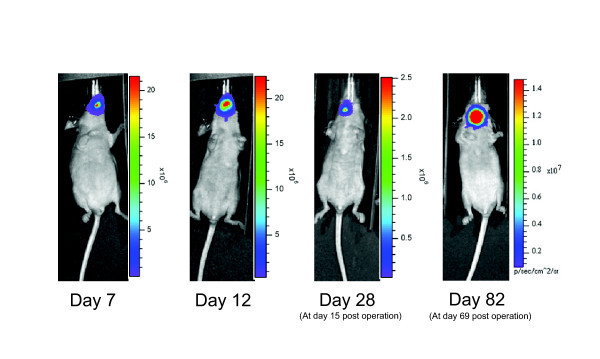
**Bioluminescence imaging of mice orthotopically transplanted with luciferase-transduced cells**. Bioluminescence imaging of mice orthotopically implanted with luciferase-transduced OSC-19 LN2-Luc cells. Mice were imaged using the IVIS 200 imaging system (Xenogen Corporation, Berkeley, CA). In brief, mice were first injected intraperitoneally with 40 mg/kg luciferin (Xenogen Corporation) and were anesthetized with 2% isoflurane (Abbott Laboratories, Chicago, IL). On day 7, mice had no cervical lymph node metastases. By day 12, mice showed a progression of tumor spread from the tongue to the cervical lymph nodes. The primary tumor was removed surgically on day 13 to allow for increased animal survival and to allow time for the lymph node metastases to grow and further develop. On day 82, increased intensity with cervical lymph node metastasis was observed.

We have used luciferase-transduced SCCOT cell lines and orthotopic animal models to visualize SCCOT growth and metastasis *in vivo*. SCCOT cells were retrovirally infected with green fluorescent protein and the firefly luciferease gene with pBMN-I-lucoferase-GFP, and we analyzed the primary tumor growth and regional metastasis of this orthotopic mouse model of SCCOT using the IVIS 200 imaging system (Xenogen Corporation, Berkeley, CA) [30,42]. In this way, we were able to monitor regional metastasis and quantify the growth of primary SCCOT using localized photon emission. To monitor the behavior of cervical lymph node metastasis, the partial glossectomy technique can be useful for decreasing emission from the primary tumor and providing longer animal survival times, giving metastases more time to develop (Figure 2). Henson et al. have successfully used the luciferase gene in *in vivo *in an orthotopic floor of mouth model to visualize local-tumor growth and metastasis [43]. While the technique with luciferease-labeled cancer cells has only been reported by a few investigators to date, it appears to be useful for monitoring the response to targeted therapeutic agents [30]. This technique will enable investigators to better study the treatment of regional metastasis of HNSCC *in vivo*.

## Conclusion

In this review, we describe subcutaneous and orthotopic models of head and neck cancer, which can be useful tools for investigating the tumor biology and treatment of head and neck cancers. Overall, orthotopic xenograft models have several advantages over ectopic subcutaneous xenograft models. In addition, the use of small-animal imaging systems for detecting tumors and metastases expands the utility of these models for studies on the mechanisms of tumor progression and metastasis, as well as for preclinical models of novel therapeutic regimens.

## Competing interests

The authors declare that they have no competing interests.

## Authors' contributions

DS and JNM wrote the manuscript. Both the authors read and approved the final manuscript.
